# Development of an Online Resource for People Bereaved by Suicide: A Mixed-Method User-Centered Study Protocol

**DOI:** 10.3389/fpsyt.2021.770154

**Published:** 2021-12-21

**Authors:** Edouard Leaune, Laurène Lestienne, Pierre Grandgenèvre, Margot Morgiève, Guillaume Vaiva, Maxime Vieux, Benoît Chalancon, Nathalie Laplace, Julie Haesebaert, Emmanuel Poulet

**Affiliations:** ^1^Centre Hospitalier Le Vinatier, Bron, France; ^2^INSERM, U1028, CNRS, UMR5292, Lyon Neuroscience Research Center, Psychiatric Disorders: from Resistance to Response – PSYR2 Team, Lyon, France; ^3^Groupement d'étude et de prévention du suicide, Saint-Benoît, France; ^4^Univ. Lille, INSERM, CHU Lille, U1172 - LilNCog - Lille Neuroscience & Cognition, Lille, France; ^5^Centre de Recherche Médecine, Sciences, Santé, Santé Mentale, Société (Cermes3), UMR CNRS 8211, Unité INSERM 988-EHESS-Université Paris Descartes, Paris, France; ^6^Centre National de Ressources & Résilience pour les Psychotraumatismes (Cn2r Lille Paris), Lille, France; ^7^Interlude Santé, Brignais, France; ^8^EA 7425, HESPER Health Services and Performance Research—Claude Bernard Lyon 1 University, Université de Lyon, Lyon, France; ^9^Department of Emergency Psychiatry, University Hospital Edouard Herriot, Hospices Civils de Lyon, Lyon, France

**Keywords:** bereaved, user-centered design, digital, grief after suicide, suicide

## Abstract

**Introduction:** Suicide bereavement is known to be highly distressing and is frequently associated with mental health problems. Despite high-level of need regarding mental and physical health, people bereaved by suicide display low level of help-seeking and perceived support in the aftermath of the loss. The lack of accessibility and reliability of face-to-face counseling resources is notably reported by suicide survivors. Online resources can enhance early access to help and support for people bereaved by suicide. The primary objective of the study is to design and implement an innovative and adaptive online resource for people bereaved by suicide according to their needs and expectation regarding online solutions dedicated to suicide bereavement.

**Methods:** The ESPOIR_2_S study is a mixed-method user-centered study. ESPOIR_2_S seeks to build the resource from the perspectives and needs of both people bereaved by suicide and professionals or volunteers working in the field of postvention. The Information System Research (ISR) Framework is used to guide the design of the study through a 3-step research cycle. The structure of the ESPOIR_2_S study relies on a simultaneous collection of qualitative and quantitative data which will be collected and analyzed during (a) the *Relevance cycle* through an online questionnaire and focus groups; (b) the *Design cycle* through focus groups; and (c) and the *Rigor cycle* through an online questionnaire and semi-structured interviews. The user-centeredness will be ensured by the active participation of people bereaved by suicide, members of associations for bereaved people and professionals of postvention.

**Discussion:** The mixed-method and user-centered design of the ESPOIR_2_S study will offer an in-depth collection of the needs and expectation of suicide survivors regarding online resources. Through the implementation of an adaptive online solution, we aim to enhance the access to help and support for suicide survivors which are highly correlated with well-being and recovery.

## Introduction

For every occurring suicide, 135 people are exposed, and 6–14 consider themselves bereaved (1). A recent meta-analysis (2) reported a lifetime prevalence of 21.8% for exposure to suicide in the general population and a lifetime prevalence of 3.9% for exposure to suicide in the family. According to the “continuum of survivorship” developed by Cerel et al. (3), the distinction between people “exposed” to suicide, those “affected” by suicide death, “short-term suicide bereaved” and “long-term suicide bereaved” is critical. Suicide bereavement is indeed known to be highly distressing (4, 5) and is frequently associated with mental health problems, including suicidal ideation and behaviors, complicated grief, acute and posttraumatic stress disorders, mood and anxiety disorders (6) and substance use (7). Notably, the prevalence of complicated grief (25–43%) and suicidal ideation (14–49%) are particularly high (6). Specific grief processes have been identified in suicide bereavement, including the violence of death, guilt and shame, stigma, loss of meaning and a high intensity of psychological distress. The social impact is also deleterious, with a high level of perceived stigma associated with social withdrawal (8) and dropping out of education and work (9). Physical adverse outcomes, especially pain, cardiovascular disease, hypertension, diabetes and chronic obstructive pulmonary disease, are also frequent causes of short- and long-term disability in people bereaved by suicide (10).

Despite the deleterious impact of suicide bereavement on mental health, survivors can experience recovery in the months and years following the loss of their loved one (11–13), especially when receiving effective social support (14). Offering effective social support to people bereaved by suicide is thus a critical challenge to improving the mental health and well-being of suicide survivors. In a recent systematic review of 32 studies, peer support by other suicide survivors was shown to be associated with reduced grief symptoms and increased well-being and recovery (15).

Despite a high level of need regarding mental and physical health, people bereaved by suicide display a low level of help-seeking and perceived support in the aftermath of the loss (16). The lack of accessibility and reliability of face-to-face counseling resources is notably reported by suicide survivors. Additionally, according to a recent meta-analysis, more than half of all bereaved people use digital resources for their grief work (17). These online resources are diverse and include informative websites about grief and loss, memorial websites, online support groups, and online therapy and counseling (18). The most commonly used resources are online support groups, social media and memorial websites (18). Online resources can help to overcome obstacles to receiving support *via* their geographic independence, offering support 24/7 and easier access than off-line resources (19). Moreover, some web-based support is anonymous, which may overcome the shame and stigma experienced by some bereaved individuals.

Postvention refers to the activities developed in the aftermath of a suicide to prevent negative health outcomes and facilitate recovery among the bereaved (20). According to a recent systematic review (19), online resources can enhance early access to help and support for people bereaved by suicide, for whom the need for early and pro-active postvention interventions has recently been reported (21). When comparing participants in online support groups to face-to-face groups, Feigelman et al. (22) found that users of Internet groups were significantly younger, more frequently women, and less educated; additionally, they had lower incomes, were less religious, and were more often divorced or separated and living alone. They were more recently bereaved and had experienced more stigmatizing responses from their family and other relatives. As such, this finding indicates that online resources may be of particular interest to reach people who might otherwise not be able to access the support they need because of personal and structural barriers. These resources are also considered by bereaved people as an addition rather than a substitute for other sources of support (19). They are mainly used to seek and share support, find and share information, memorialize the loved one and for meaning making. In terms of grief reactions, most users of online resources report the importance of finding information about mourning and discussing grief-related topics, which could be taboo or stigmatized topics offline. Despite a shortage of evidence of the effectiveness of online resources, most studies report perceived benefits (23–28), such as the possibility of using the resources around the clock and discussing grief-related topics without being judged. Kramer et al. (26) reported a significant increase in well-being and a significant decrease in depressive symptoms after 12 months of use of an online forum. Westerlund et al. (28) found that online support group activity was significantly associated with satisfaction regarding psychosocial health (*p* < 0.001), while memorial website activity showed a tendency to have a negative association (*p* = 0.05). Having online access to support from other people bereaved by suicide is also highly valued, which is in line with what is known about the experienced value of peer suicide bereavement support (15). Finally, some potentially negative aspects of web-based resources are reported as concerns rather than actual experiences. The need for sooner and more widespread access to resources is reported, as well as the risk of spending too much time on the Internet or becoming overly attached to it (23, 24, 27). While the important need for support for people bereaved by suicide is often not fulfilled, online resources are a much-needed addition to available, mostly in-person, resources.

While France shows one of the highest suicide rates in Europe (29), no institutional evidence-based online resource for people bereaved by suicide is currently available. Hospital-based consultation centers led by psychiatrists and/or psychologists and charities created by bereaved individuals exist, but the lack of accessibility for those in need is critical. Implementing an innovative and adaptative evidence-based online resource for French people bereaved by suicide is thus urgently needed (a) to offer the easiest access to information, help and support, (b) to reinforce prevention of deleterious impact of suicide bereavement on mental health, and (c) to inform the best practices to implement online resources in the field of mental health. The user-centered design of implementation research is critical to develop evidence-based solutions and to improve their usability and translation into practice settings (30). Moreover, previous research demonstrated that participating in postvention studies is helpful for people bereaved by suicide, as it improves social support, altruism, and personal growth (31).

According to this need for psychosocial change, the primary objective our study is to build and implement an online resource for people bereaved by suicide through a user-centered design. The secondary objectives are to collect the needs and expectations of people bereaved by suicide regarding online resources and to evaluate the acceptability and the perceived benefits and/or adverse effects of the designed solution.

## Methods

### Study Objectives

The primary objective of the ESPOIR_2_S study is to design and pilot-test an innovative and adaptive online resource for people bereaved by suicide according to their needs and expectations regarding online solutions dedicated to suicide bereavement.

The secondary objectives are to (1) collect the needs and expectations of people bereaved by suicide regarding online resources dedicated to suicide bereavement and (2) evaluate the acceptability and the perceived benefits and/or adverse effects of the designed solution.

### Study Design

**Figure 1:**
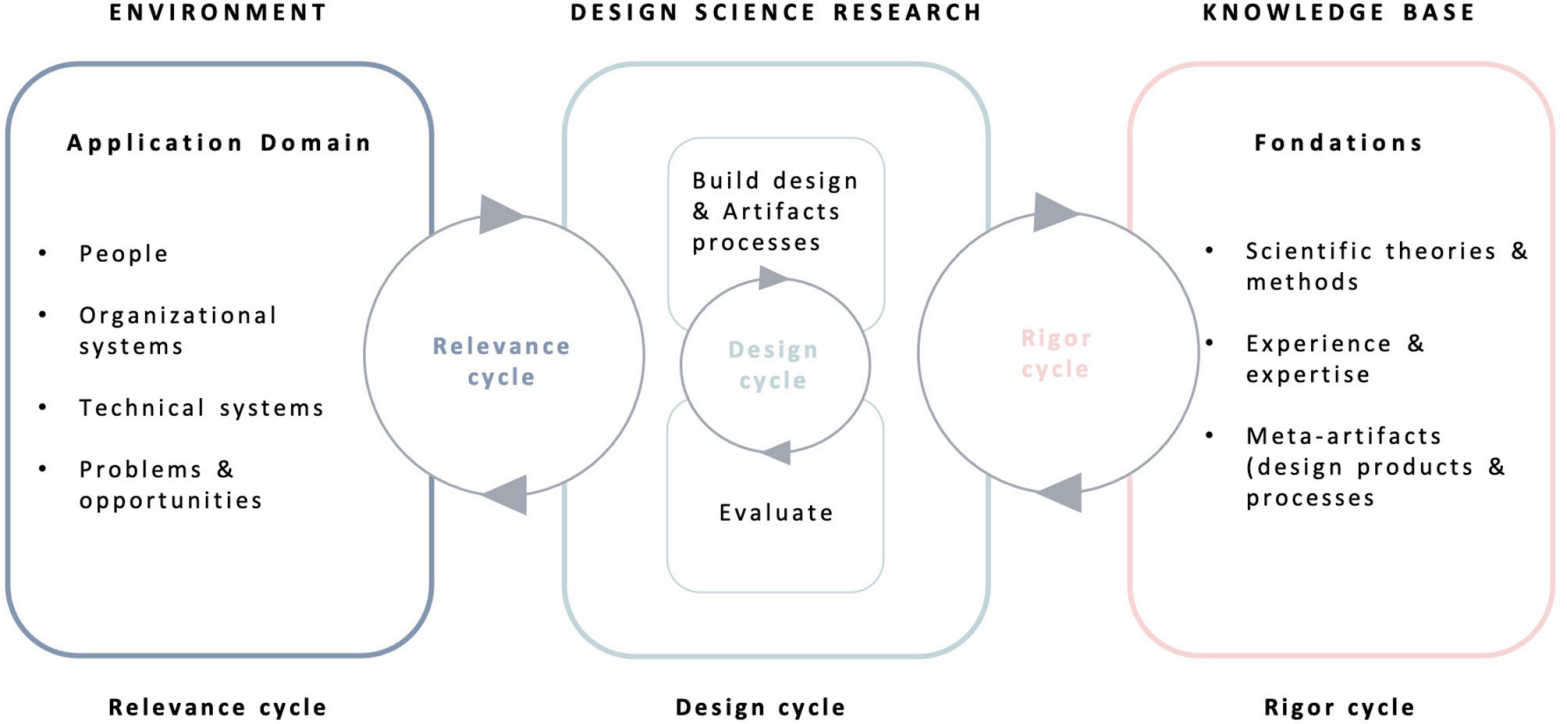
The information system research framework.

**Figure 2:**
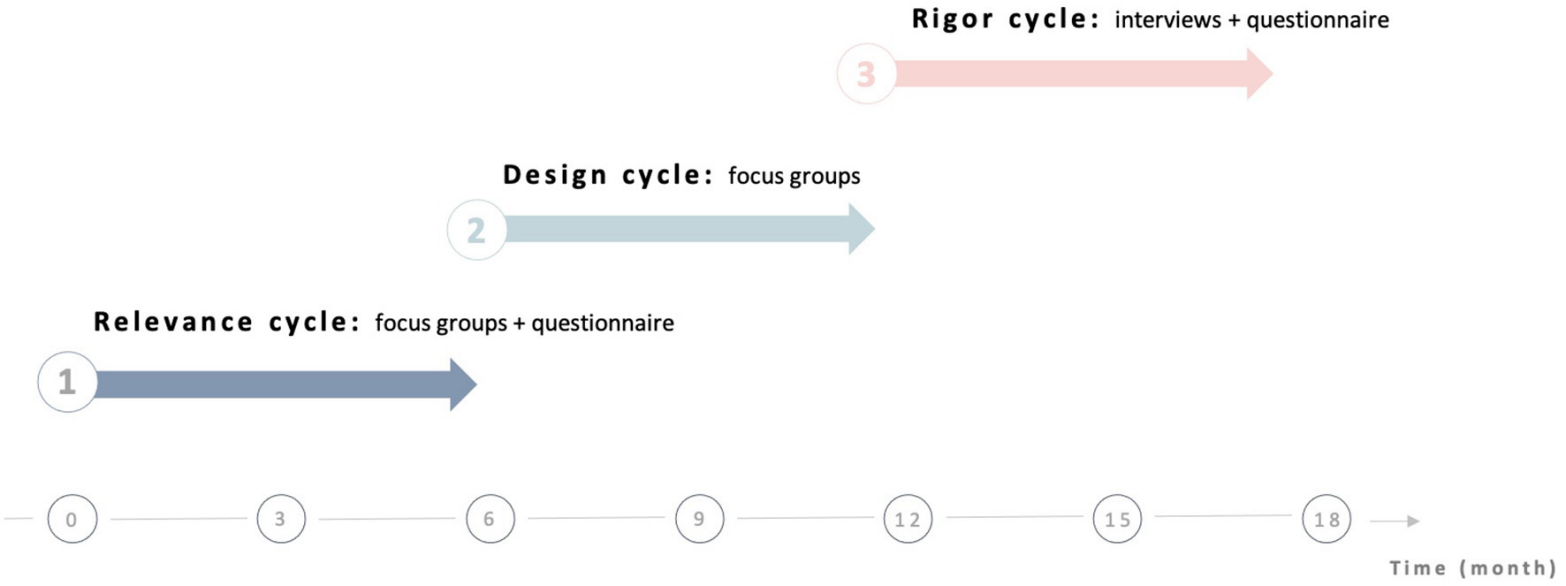
Schematic overview of the ESPOIR_2_S study.

The ESPOIR_2_S study is a mixed-method collaborative and participatory user-centered study. ESPOIR_2_S seeks to build resources from the perspectives and needs of both people bereaved by suicide and professionals or volunteers working in the field of postvention. According to the *Medical Council Research* guideline for the development of complex intervention (32), our study is situated as a pilot project of development/design/evaluation. Thus, we used the Information System Research (ISR) framework to guide the design of the study (32). The ISR framework (Figure 1) employs various design processes to build a product or design an artifact such as a mental health online resource. According to a recent study (33), a 3-stage research cycle is planned, including (1) the *relevance cycle*, (2) the *design cycle* and (3) *the rigor cycle* (Figure 2).

#### Mixed-Method Evaluation

Mixed-method studies have been shown to be effective in evaluating the implementation of new prevention and intervention programs in mental health (34). A mixed-method design focuses on collecting, analyzing and merging both quantitative and qualitative data into one study. According to the taxonomy of mixed-method studies described by Palinkas et al. (34), the structure of the ESPOIR_2_S study relies on a simultaneous collection of qualitative and quantitative data. The two datasets will be collected in complementarity, e.g., qualitative data will be used to provide depth of understanding, and quantitative data will be used to provide breadth of understanding. The process of data analysis will be performed by merging the two datasets. The ESPOIR_2_S study is thus designed as a simultaneous complementary merging mixed-method study.

#### User-Centeredness

The participation of people bereaved by suicide in the three cycles of the study will ensure the user-centeredness of both the resource development and the research process. Their active participation throughout the entire research and design/implementation process will offer a unique means to develop an innovative and adaptive resource that meets the needs and expectations of people bereaved by suicide. The coproduction of knowledge on suicide bereavement and online resources will allow users to use and share their experiential knowledge, skills and expertise with researchers and other participants (35). Creating, through interaction, an empowering culture of practices within the team will ensure that the users feel free to fully express themselves and to fully contribute to the research process (36). The participatory and collaborative design of our study will be guaranteed by the creation of an environment of equality, equity and empowerment.

#### Inclusion and Exclusion Criteria

French adults bereaved by suicide are eligible to participate in the 3 stages of the ESPOIR_2_S study. Professionals or volunteers working in postvention units or charities dedicated to bereavement are also eligible for participating in the Stages 1 and 2 (focus groups). People under 18 and people bereaved by other causes than suicide will be excluded of the study.

#### Data Collection

According to the mixed-method research design of the study, quantitative and qualitative data will be simultaneously and complementarily collected through a 3-stage process to obtain an in-depth evaluation of the implementation of the online resource (Figure 2). Quantitative and qualitative data will be collected and analyzed during (a) the *relevance cycle* through an online questionnaire and focus groups, (b) the *design cycle* through focus groups and (c) the *rigor cycle* through an online questionnaire and semi-structured interviews.

### Stage 1—Relevance Cycle

#### Objective

The *relevance cycle* aims to understand the environment of the end user by determining requirements through a mixed-method approach combining the quantitative and qualitative collection of data. The needs and expectations of people bereaved by suicide, professionals and volunteers working in the field of postvention will be collected through a series of focus groups and an online questionnaire.

#### Methods

The *26-item online questionnaire* was built for the study according to a recent systematic review of online resources for suicide bereavement (19) and will be hosted on the website *LimeSurvey*. The questionnaire collects sociodemographic characteristics (age, gender, socioeconomic status, relation to the deceased) and evaluates four dimensions: (1) perceived needs regarding suicide bereavement (social support, professional counseling, peer support, meaning-making); (2) use of online resources associated with the suicide loss [frequency, type(s) of resources, reasons for using online resources, satisfaction with the resources]; (3) needs and expectations regarding the development of an online resource for people bereaved by suicide (types of resource, technologies, typology of use); and (4) personal propositions regarding the development of an online resource for people bereaved by suicide (type of resource, technologies, usability). We measured that the participation of 385 respondents would permit us to estimate a proportion with a 95% confidence interval and a 5% error margin, as determined in a previous study (37). Due to the method of dissemination of the online survey by convenience sampling, we were not able to define the size of our source population, so we chose the highest estimate of 385 as sample size for the online survey.

**Figure 3:**
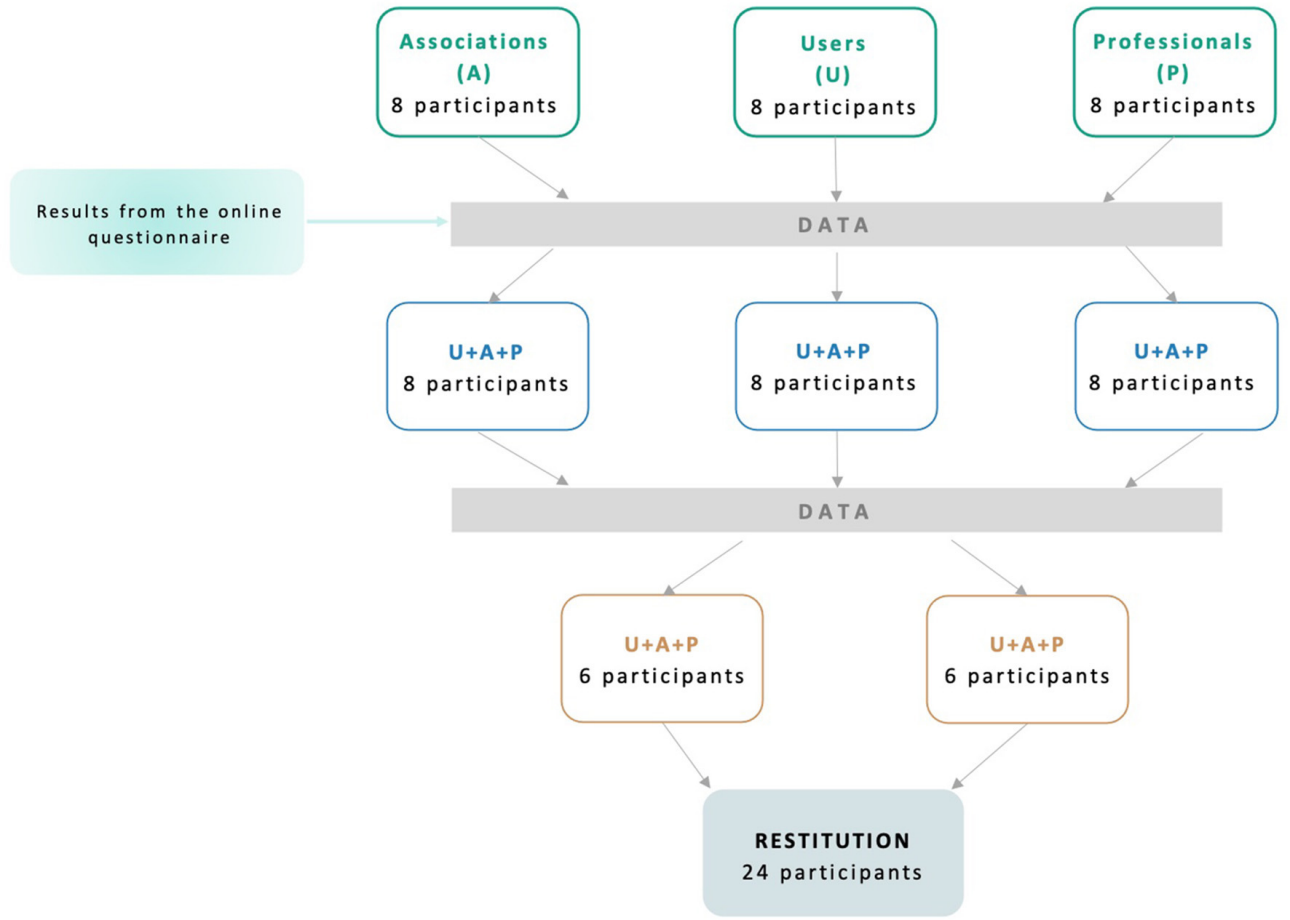
Schematic overview of the relevance cycle.

A total of eight *2-hour focus groups* will be performed in a 3-step model (Figure 3) including 3 categories of participants: people bereaved by suicide, postvention professionals, and volunteers working in the field of bereavement and postvention. During the first step, 3 independent focus groups with each type of participant will collect the needs and expectations of the participants. The second step will include 3 focus groups mixing all types of participants. The results issued from the online questionnaire and the first focus group will be presented to participants to initiate the discussion. The third step will include 2 focus groups mixing all types of participants. The results issued from the previous focus group will be presented to participants to initiate the discussion. A last step of restitution to all participants is planned to present and adjust the final results.

#### Data Collection

Individual quantitative data will be collected through the online questionnaires hosted on the web application *LimeSurvey*. The questionnaire will be sent to putative participants through social media (Facebook, Twitter) and through associations for people bereaved by suicide. A purposive snowballing sampling will be used to send the link to the online questionnaire to a maximum of putative participants through social media posts and emails.

Each focus group will last ~2 h and will include 6–8 participants, according to usual recommendations on qualitative research (38). To obtain the perspective of all profiles of stakeholders, we will adopt a maximum variation recruitment process to constitute our focus groups (age, gender, relation to the deceased). To facilitate the collaborative process, each focus group will be led by two researchers (a facilitator and an observer) and organized through the following process: (a) work objectives, (b) emerging needs and expectations, (c) hypotheses and concepts, (d) putative solutions, and (e) conclusions. A guide written by the pluriprofessional team and users bereaved by suicide will be used to guide the focus group. The facilitator will engage the focus group through the following question “*What are your expectations about an online resource for people bereaved by suicide?*” and then dynamize the discussion with the participants through probing questions on the content, design or limitation of the resource. The observer will collect the verbal and non-verbal data obtained from the discussions and interactions among the participants. The content of the focus group will be recorded and anonymized. The needs and expectations of people bereaved by suicide will be collected and synthesized to build a design brief for online resources. The data collected during the Relevance Cycle will inform the design brief regarding the expected technologies (website, mobile app, online forum…) and the contents of the resource (testimonies, information on suicide prevention and bereavement, live chat...).

### Stage 2—Design Cycle

#### Objective

The *design cycle* aims to produce online resources by compiling qualitative and quantitative data collected during the *relevance cycle* through an iterative process of building and evaluating online resources.

#### Methods

Two to five focus groups will be performed with people bereaved by suicide, members of associations for bereaved people, postvention professionals and web designers from a start-up specialized in *e*-health solutions. Each focus group will be led by two researchers, as during the *relevance cycle*, and will lead to the redaction of specifications and/or adjustments of the resource, based upon which the web designers will progressively and iteratively design a prototype of the online resource. The iterative feedback of participants on the resource and its final validation will ensure the progressive design of an online resource adapted to the needs and expectations of suicide survivors and stakeholders.

#### Data Collection

Each focus group will last ~2 h and will include 6–8 participants, being organized through the same processes as during the *relevance cycle*. Again, the facilitator will engage the focus group by presenting the first draft of the resource built by the web designers and then dynamize the discussion through stimulus questions about the perception of the resource by the participants (usability, design, contents, interactivity), while the observer will collect the data obtained from discussions and interactions. A total of 16 people will participate in the focus groups. The content of the focus group will be recorded and anonymized. A pilot of the online resource will be designed and built at the end of the *design cycle*, before its evaluation during the *rigor cycle*.

### Stage 3 - Rigor Cycle

#### Objective

The *rigor cycle* aims to evaluate the acceptability and perceived effectiveness of the online resource (pilot test).

#### Methods

A total of 30 people bereaved by suicide will be recruited through the mailing lists of associations dedicated to bereavement to test and evaluate the online resource. This sample size is not defined by statistical assumptions but by the feasibility of inclusion in a pilot-test over a reduced time period. The association of a quantitative and qualitative analysis of acceptability will enable us to identify strengths and weaknesses of our online resources to proceed (or not) further with improvements and with a larger scale effectiveness evaluation study. The French version of the *System Usability Scale* (SUS) (39) will be used to assess (a) the satisfaction regarding the resource and (b) the acceptability of its design and content. Semi-structured interviews will also be performed with the participants to qualitatively assess the accessibility and perceived effectiveness of the resource.

#### Data Collection

The 20-item online questionnaire will be hosted on the web application *LimeSurvey*. Sociodemographic data (gender, age, duration of bereavement, relation with the deceased), usability of the resource and perceived benefits for the participants will be collected. The SUS is a 10-item Likert-scale questionnaire that provides a global view of the subjective assessment of a system's usability (39). The SUS scores have a range of 0–100 (score ranges from 0 to 10 for each item) (39). A score above 70 indicates good acceptability of a resource and a score above of 80 indicates a very good acceptability (40). We will conclude that acceptability is reached if at least 80% of respondents rated the acceptability on the SUS above 80. Acceptability will also be assessed during semi-structured interviews to better understand how the online resource might be improved. The final evaluation of acceptability will be based on the combination of quantitative SUS results and qualitative results. Each semi-structured interview will last between 30 and 60 min and will be led by two researchers. The participants will be questioned about their perception of the online resource (satisfaction, usability, acceptability) and the putative modifications that they may recommend.

A total of 30 people bereaved by suicide will participate in the *rigor cycle*. The final version of the online resource will be designed and built at the end of the rigor cycle, before its implementation.

### Data Analysis

Qualitative results will be combined with quantitative results to both provide a detailed overview regarding acceptability of the online resource.

#### Quantitative Analysis

Data manipulation and analyses will be performed using R software (R 3.4.1). Categorical variables will be summarized using numbers and percentages, and quantitative variables will be described using either means and standard deviations or medians and interquartiles. For Stage 1, number of respondents, number of completed questionnaires, characteristics of respondents (age, gender, socioeconomic status, and relation to the deceased) and responses to the questionnaire will be analyzed using descriptive statistics. Subgroup analyses of responses according to age category (defined according to the distribution of age of the respondents), gender, and relation to the deceased will be conducted. A report summarizing the results will be produced and presented during the focus groups. For Stage 3, the characteristics of the included population will be described. The main outcome criteria will be the score at the SUS. The SUS will be described as a continuous variable by its mean ± SD, median and interquartile range. SUS will also be dichotomized: SUS > 80 (very good usability) vs. SUS < 80 (moderate/poor usability) (40). A multivariate analysis will be conducted using a multivariate linear model to identify which factors are associated with highest usability scale. The dependent variable will be the SUS score, independent variables will be age, gender, socioeconomic status and relationship with the deceased. All tests will be two-tailed and the statistical significance threshold will be set at 5%.

#### Qualitative Analysis

Based on the narratives of the focus groups or semi-structured interviews, a content analysis will be performed, with several chronological phases (reanalysis, operation of equipment and interpretation) (41). According to a new method developed by Renz et al. (42), two data analyses (i.e., manual and computer-based) will be combined to enhance the trustworthiness of the results. The first method is based on a manual content analysis (43). Two authors (EL, LL) will first look at the apparent messages through a repeated reading of the transcripts to achieve immersion and obtain a sense of the whole. In addition, this first reading will allow us to define the thematic and formal categories relevant for later coding speeches. Units of meaning will then be independently identified, categorized and put into relation to identify axes of transversal meanings. This process will allow us to classify the elements and to emit a simplified representation of the raw data. The second method will use a computer-based content analysis through NVivo software and will be performed by another author (MV). NVivo is a computer-assisted qualitative data analysis software that allows for qualitative inquiry beyond the coding, sorting and retrieval of data (44). The benefits of using NVivo are outlined in terms of facilitating teams of researchers to systematically and rigorously synthesize qualitative data.

### Limitation of Biases

The following main bias limitations will be used in the ESPOIR_2_S study: (a) triangulation, (b) saturation, and (c) participatory approach.

*Triangulation* refers to the use of multiple methods or data sources in qualitative and mixed-method research to develop a comprehensive understanding of phenomena (45, 46). Denzin (47) and Patton (45) identified the following four types of triangulation: (a) method triangulation, (b) investigator triangulation, (c) theory triangulation and (d) data source triangulation. The four types of triangulation will be used in the ESPOIR_2_S study through the mixed-method design (method and data source triangulation) and the involvement of users and researchers from different disciplines and theoretical backgrounds (theory and investigator triangulation). Intramethod triangulation will also be added through the two methods of content analysis previously described.

*Saturation* is used in qualitative designs as a criterion for discontinuing data collection and analysis (48). According to the taxonomy developed by Saunders et al. (48), the following four types of saturation are defined: (a) theoretical saturation (i.e., the development of theoretical categories), (b) inductive thematic saturation (i.e., the emergence of new codes or themes), (c) *a priori* thematic saturation (i.e., the degree to which identified codes or themes are exemplified in the data), and (d) data saturation (i.e., the degree to which new data repeat what was expressed in previously collected data). The saturation used in the ESPOIR_2_S study will focus on data saturation and inductive thematic saturation.

The *participatory approach* is an effective method to limit the misinterpretation of the collected data by researchers. The involvement of people bereaved by suicide in the research process promotes the ability to adjust the designed solution as well as the conclusions issued from the research through their direct comments on the results.

### Funding Sources and Ethical Approval

The ESPOIR_2_S study is funded by the *Scientific Research Committee from the Centre Hospitalier le Vinatier* (funding number CSRN05) and by the *National Institute for Public Health Research* (Institut de Recherche en Santé Publique – funding number IRESP-RSP2020- 230791). The study received ethical approval from the Ethical Review Board of The University Claude Bernard Lyon 1 (registration number 2021-01-12-04).

## Discussion

Suicide bereavement is known to be highly distressing and is frequently associated with mental health problems. However, people bereaved by suicide display low levels of help-seeking and perceived support in the aftermath of the loss. Online resources dedicated to suicide bereavement offer unique opportunities to enhance early access to help and support for people bereaved by suicide. ESPOIR_2_S is the first study aimed at developing and pilot-testing an innovative and adaptive online resource for people bereaved by suicide. As counseling and social support have been proven to be associated with better well-being and recovery, effective access to resources to fulfill the needs of suicide survivors represents a key component of postvention. The feasibility of the study and the development/implementation of the online resource is high, as it relies on a national network of both clinical and research suicide prevention centers and charities dedicated to people bereaved by suicide. The mixed-method and user-centered design of the ESPOIR_2_S study will offer an in-depth collection of the needs and expectations of suicide survivors regarding online resources, and thus allow us to design and implement a solution that adequately meets the identified needs.

Our study protocol has several limitations. First, ESPOIR_2_S will be performed in France, which may impede the generalizability of our results. Indeed, grief processes and support for those who are bereaved are known to be shaped by the cultural context (49). However, a recent systematic review reported similar patterns of use and effectiveness of online resources for people bereaved by suicide across countries (19). Second, the evaluation of the designed online resource relies only on the acceptability and perceived benefits of a small number of participants (*n* = 30). Thus, after the design study, a second longitudinal study will be performed and include a larger and representative sample. This will allow us to better understand the effectiveness of online resources for suicide bereavement. Third, selection bias may hamper the generalizability of our results, as users who will participate in the study may show better mental health or socioeconomic status than the global population of people bereaved by suicide. However, large participation in the online questionnaire is expected in the *relevance cycle*, so the representativeness of the sample should be ensured.

Despite these limitations we expect that this study will offer an opportunity to develop an innovative and adaptive online resource for people bereaved by suicide. The mixed-method and user-centered design of the ESPOIR_2_S study is innovative as qualitative and quantitative data will be collected and analyzed throughout an original research process based on the active participation of users. Through the implementation of an online solution for people bereaved by suicide adapted to their needs, we aim to enhance access to help and support, as both are highly correlated with well-being and recovery.

## Data Availability Statement

The original contributions presented in the study are included in the article/supplementary material, further inquiries can be directed to the corresponding authors.

## Ethics Statement

The ESPOIR2S study received ethical approval from the Ethical Review Board of the University Claude Bernard Lyon 1 (Registration No. 2021-01-12-04). The patients/participants provided their written informed consent to participate in this study.

## Author Contributions

EL and LL: conceptualization, methodology, investigation, and writing original draft. PG and MM: conceptualization, methodology, and writing—review and editing. MV, BC, and NL: conceptualization and writing—review and editing. JH: conceptualization, methodology, supervision, and writing—review and editing. GV and EP: conceptualization, supervision, writing—review and editing and validation. All authors have read and approved the manuscript.

## Funding

The ESPOIR_2_S study was funded by the *Scientific Research Committee from the Centre Hospitalier le Vinatier* (funding number CSRN05) and by the *National Institute for Public Health Research* (Institut de Recherche en Santé Publique – funding number IRESP-RSP2020- 230791).

## Conflict of Interest

The authors declare that the research was conducted in the absence of any commercial or financial relationships that could be construed as a potential conflict of interest.

## Publisher's Note

All claims expressed in this article are solely those of the authors and do not necessarily represent those of their affiliated organizations, or those of the publisher, the editors and the reviewers. Any product that may be evaluated in this article, or claim that may be made by its manufacturer, is not guaranteed or endorsed by the publisher.
